# Quantification of sensitivity and resistance of breast cancer cell lines to anti-cancer drugs using GR metrics

**DOI:** 10.1038/sdata.2017.166

**Published:** 2017-11-07

**Authors:** Marc Hafner, Laura M. Heiser, Elizabeth H. Williams, Mario Niepel, Nicholas J. Wang, James E. Korkola, Joe W. Gray, Peter K. Sorger

**Affiliations:** 1 HMS LINCS Center, Laboratory of Systems Pharmacology, Department of Systems Biology, Harvard Medical School, Boston, MA 02115 USA; 2 MEP LINCS Center, Department of Biomedical Engineering and Oregon Center for Spatial Systems Biomedicine, Oregon Health & Science University, Portland, OR 97239 USA

**Keywords:** Pharmacodynamics, Breast cancer, High-throughput screening

## Abstract

Traditional means for scoring the effects of anti-cancer drugs on the growth and survival of cell lines is based on relative cell number in drug-treated and control samples and is seriously confounded by unequal division rates arising from natural biological variation and differences in culture conditions. This problem can be overcome by computing drug sensitivity on a per-division basis. The normalized growth rate inhibition (GR) approach yields per-division metrics for drug potency (*GR*_*50*_) and efficacy (*GR*_*max*_) that are analogous to the more familiar *IC*_*50*_ and *E*_*max*_ values. In this work, we report GR-based, proliferation-corrected, drug sensitivity metrics for ~4,700 pairs of breast cancer cell lines and perturbagens. Such data are broadly useful in understanding the molecular basis of therapeutic response and resistance. Here, we use them to investigate the relationship between different measures of drug sensitivity and conclude that drug potency and efficacy exhibit high variation that is only weakly correlated. To facilitate further use of these data, computed GR curves and metrics can be browsed interactively at http://www.GRbrowser.org/.

## Background & Summary

The prioritization of compounds for lead discovery, identification of pharmacogenomic associations, and study of cellular machinery requires reproducible and accurate data on drug response in cultured cell lines. In the case of anti-cancer drugs, which act by slowing cell proliferation or increasing the rate of cell death, the traditional approach is to expose cells to a drug over a range of concentrations, measure the number of cells at the end of a fixed period of time (typically 3 days) and then compare that number to the number of cells in a vehicle-only control. The ratios are usually fitted to a sigmoidal curve from which it is possible to compute *IC*_*50*_, the drug concentration at which cell count is half the control value, which is a traditional measure of potency, and *E*_**max**_, the maximum effect at the highest concentration tested, which is a measure of efficacy. Although in use for many decades, this calculation is sensitive to the number of cell divisions taking place over the course of the assay, which differ when cell division rates vary. As a consequence, it is possible for *IC*_*50*_ values for a single drug in a single cell line to vary 100-fold or more simply as a consequence of exogenously imposed changes in the rate of division^1,2^. Natural cell division rates vary from one cell line to the next, often in a systematic manner depending on tissue of origin^2,3^, media composition, culture conditions, and plating density^1^. Because division rate is a powerful confounder in the calculation of *IC*_*50*_ and *E*_*max*_ values, and because it is often poorly controlled, we speculate that it is a contributor to the observed irreproducibility of drug dose-response data^2–5^.

The normalized growth rate inhibition (GR) method recently introduced by Hafner *et al.*^1,2^ corrects for variation in division rates by estimating the magnitude of drug response on a per-division basis. The GR value in the presence of drug at concentration *c* is *GR(c)*=*2*^*k(c)/k(0)*^*−1* where *k(c)* is the growth rate of drug-treated cells and *k(0)* is the growth rate of untreated control cells. Growth rates can be estimated using time course data or, as in this paper, the number of cells prior to and at a fixed time after the addition of drug. Metrics of drug potency, *GR*_*50*_, and efficacy, *GR*_*max*_, can then be calculated from the dose-response curve. The sign of the GR value is related directly to response phenotype: negative values reflect cytotoxicity, a value of 0 corresponds to complete cystostasis, and positive values reflect partial growth inhibition. GR calculations are described in detail at http://www.GRbrowser.org/, and a detailed method for performing the calculations with web-based software and an R pipeline is also available^6,7^.

The datasets presented here make use of drug dose-response data that have been described previously^8,9^ as well as substantial new data and comprise, after filtering low-quality data points, GR-based dose-response data for 71 breast cancer cell lines from all three major subtypes of breast cancer (*HER2* amplified, hormone receptor positive, and triple negative) and 107 perturbagens (104 small molecule drugs and 3 therapeutic antibodies). We found that low growth was a good criterion for filtering out data with high relative error, as judged by standard error of the mean (SEM) for GR values, presumably because: (a) a very low division rate (fewer than one division per 72 h) is an indicator that the experimental setup is not optimized for the growth of a particular cell line; (b) fixed error in Cell Titer-Glo (CTG) assays makes measurement of smaller differences in cell number between control and experimental samples unreliable; and (c) partial cell cycle synchronization introduced during plating of cells with division times longer than the assay duration violates the assumption in GR calculations that growth is homogenous and constant^10^. As a quality control filter, we therefore eliminated from further consideration all samples in which the vehicle-only controls underwent too few divisions. Despite the need to filter out some data, the 4,700 unique cell line/perturbagen pairs described in this paper represent one of the largest collections of growth-corrected drug-response measurements currently available.

## Methods

### Cell culture and treatment

The original dataset [DS0, Data Citation 1] comprised the effects of 139 perturbagens (small molecules and therapeutic antibodies) on 73 breast cancer cell lines, some in multiple cell growth conditions, determined by plating cells at a density such that they remained in logarithmic growth during the assay^11^. This required adjusting passage and plating conditions for each line.

Cells were allowed to attach overnight onto 96-well plates before being treated in technical triplicate for 72 h with nine doses of each perturbagen in a 1:5 serial dilution. The concentration range was optimized on six cell lines, and, for most cell line/perturbagen pairs, the highest concentration tested fell between 33 μM and 167 μM.

### Cell count

Cell count was estimated using the Cell Titer-Glo (CTG) Luminescent Cell Viability Assay (Promega) after three days in drug (the endpoint of the assay). Cell count was also measured in untreated plates at the time of treatment. The raw CTG assay readout values were considered proportional to the number of viable cells and used as surrogates for cell count values, which is a widely used assumption with some caveats^12^. A background CTG value was obtained from wells containing culture medium but no cells (values between 35 and 250). Dataset DS0 [Data Citation 1] comprises all raw CTG values.

### Cell count normalization and GR values

Dataset DS1 [Data Citation 2] comprises data for the 107 perturbagens (104 small molecules and 3 therapeutic antibodies) and 71 cell lines that could be unambiguously identified (i.e. could be unequivocally matched to records in public databases such as PubChem or ATCC), representing 87% of the original dataset selected for further analysis (see Data Records). First, background CTG value was subtracted from the raw CTG values (which fell between 20 and 10,000) in drug-treated wells. Background-corrected values below 1 or negative, which represented <0.1% of the data, were set to 1 to eliminate artefacts from near-background CTG values. Then, we calculated the following values:

–*x(c)*, a robust average of three technical replicates for the background-subtracted CTG value following drug treatment at concentration *c*; *x*(*c*)=mean({*x*_*i*_∈**x** | abs(log_10_(*x*_*i*_)–log_10_(mean(**x**)))<1}) where **x** is the vector of treated values.
–*x*_*ctrl*_, the robust average background-subtracted CTG value in DMSO-treated control wells from the same plate; *x*_*ctrl*_=mean({*x*_*i*_∈**x** | abs(log_10_(*x*_*i*_)–log_10_(mean(**x**)))<1.5)}) where **x** are all DMSO-treated control values.
–*x*_*0*_, the background-subtracted median value from the untreated samples (one per cell line and biological replicate) measured at the time of treatment.

Dataset DS1 [Data Citation 2] comprises the GR value for each treated condition calculated as follows^1^:

$$GR\left(c\right)=2^{\frac{\log_{2}\left(x(c)/x_{0}\right)}{\log_{2}\left(x_{ctrl}/x_{0}\right)}}-1$$

### Dose-response curves and GR metrics

GR values for a specific drug across multiple concentrations were fitted to a sigmoid curve with the equation $GR\left(c\right)=GR_{inf}+\frac{\left(1-GR_{inf}\right)\cdot GEC_{50}^{h_{GR}}}{GEC_{50}^{h_{GR}}+c^{h_{GR}}}$ using the MATLAB fitting function. More details about the procedure can be found in Hafner *et al.*^6^. Given the data points and fitted equation, we extracted the response parameters:

- ***GR***_***50***_: the primary metric of drug potency; the concentration of drug *c* at which *GR(c)*=*0.5*. If the value for *GR*_*inf*_ (see below) is above 0.5, *GR*_*50*_ cannot be defined and we set its value to +∞. If *GR*_*inf*_ is below 0.5 but the inferred *GR*_*50*_ value is half an order of magnitude (3.16-fold) above the highest tested concentration, we set the *GR*_*50*_ value to +∞ to avoid artefacts due to extrapolation (this affected 0.6% of conditions). In plots of *GR*_*50*_ values, *GR*_*50*_ values were capped at 100 μM for visualization purposes.
- ***GR***_***max***_: the primary metric of drug efficacy; the *GR* value at the highest tested dose of the drug. *GR*_*max*_ lies between –1 and 1; negative values correspond to a cytotoxic response (i.e. cell death), a value of 0 corresponds to a fully cytostatic response (no increase or decrease in cell number), and positive values less than one correspond to partial growth inhibition. *GR*_*max*_ can only be compared across drugs or cell lines when the highest tested doses are the same.
- ***GR***_***AOC***_: the integrated effect of the drug across a range of concentrations as estimated from the ‘area over the curve’. A value of 0 means no effect of the drug across the full dose-response range. *GR*_*AOC*_ can only be compared across drugs or cell lines when the dose range is the same.
- ***GR***_***inf***_: a measure of drug efficacy extrapolated to an infinitely high drug concentration as determined from the asymptote of the dose-response curve; $GR_{inf}\equiv GR(c\to \infty )$. For dose-response curves that reach a plateau under experimental conditions, the value of *GR*_*inf*_ is similar to *GR*_*max*_.
- ***h***_***GR***_: the Hill coefficient of the fitted curve; it reflects the steepness of the dose-response curve. We constrained its value between 0.1 and 5.
- ***GEC***_***50***_: an analogy to *EC*_*50*_ corresponding to the concentration of drug at half-maximal effect. *GEC*_*50*_ is relevant for drugs that have poor potency and that do not reach a GR value below 0.5. In the fitting procedure, *GEC*_*50*_ is constrained to lie within two orders of magnitude of the highest and lowest tested drug concentrations.

We excluded from further consideration cell line/perturbagen pairs in which fewer than 0.3 cell divisions (a 23% increase in cell number) were recorded for the no-treatment control over the course of a 3-day assay (this affected 8.5% of the data, the majority of which were associated with 6 specific cell lines). This was done because GR values quantify the effects of drugs on cell growth. If untreated control cultures are not growing, the response to drug is generally weak, and GR values become highly variable and biologically meaningless. In principle, it is possible to quantify the effects of highly cytotoxic drugs under these conditions using traditional *E*_*max*_ values, but fewer than 1% of the responses in the overall dataset were sufficiently cytotoxic that *E*_*max*_ values would not have been biased by the low division rate. Following filtering, we obtained 4,788 unique cell line/perturbagen pairs, the majority of which were recorded in biological duplicate for a total of 8,882 cell line/perturbagen pairs, each tested at nine doses in technical triplicate. Before averaging biological replicates when available, we further filtered the data to exclude measurements for which division time was greater than 80 h (corresponding to 0.9 division over the course of a 72 h assay). We included this cutoff based on preliminary data showing that GR values obtained from slow-growing cell lines had high uncertainty (see Technical Validation section below). The final dataset comprised 4,650 cell line/perturbagen pairs corresponding to a median of 88 perturbagens per cell line [DS2, Data Citation 2].

### Code availability

GR calculation, curve fitting, and extraction of metrics was performed using scripts published in Hafner et al.^1,6^ and found on the GitHub repo at https://github.com/datarail/gr_metrics. Other analysis was performed using MATLAB standard functions.

## Data Records

This manuscript describes 4 new datasets (DS1, DS2, DS3, DS4) that are publically available as a single data package in the Dryad Digital Repository (Data Citation 2) alongside a fifth file bundle (DS0) consisting of the reference files used to process the file containing the raw dose-response data described in Heiser et al.^8^ and Daemen et al.^9^ and additional data generated subsequently (Data Citation 1). DS1 reports relative cell counts estimated from the available CTG values as well as computed GR values and nominal division rates for all cell line/perturbagen pairs from the raw dataset for which the cell line and perturbagen reagents used could be unambiguously identified (representing 87% of the original dataset, DS0) and for which the cell line demonstrated sufficient growth over the course of the assay to exceed our cutoffs (see Methods). DS2 reports the calculated GR metrics for the same set of cell line/perturbagen pairs. DS3 and DS4 report the median, upper quartile, and lower quartile GR metrics per perturbagen and per perturbagen class, respectively, across all cell lines in DS2. DS0 consists of a downloadable zip file containing multiple reference files necessary for annotating the raw data file with reagent identifiers and perturbagen concentration details, reference file metadata in the form of data column lookup tables (LUTs), experimental metadata including a detailed protocol, and a ReadMe file. DS1, DS2, and DS3 each consist of a downloadable zip file containing a data file, data file metadata in the form of a data column LUT, experimental metadata including a detailed protocol and reagent metadata tables, and a ReadMe file. DS4 consists of a downloadable zip file containing a data file, data file metadata in the form of a data column LUT, experimental metadata including a detailed protocol, and a ReadMe file. All files are provided in their original format and, where relevant, also in a non-proprietary, preservation-friendly format (e.g. .csv, .txt). All dose-response curves and metrics corresponding to DS2 also are available for interactive viewing at http://www.GRbrowser.org.

## Technical Validation

The GR method used to perform calculations in this paper has previously been described^1^, and the code has been validated on multiple datasets. Use and installation instructions, as well as bug fixes, are available at our GitHub repo (https://github.com/datarail/gr_metrics). Methods for plating cells, adding drugs, and performing CTG assays have also been described in Heiser et al.^8^ and elsewhere. We have recently published an in-depth protocol^10^ for performing drug dose-response experiments in cell lines that includes advances in methodology not available at the time the current data were collected. We recommend that future studies follow these more recent guidelines.

**Figure 1:**
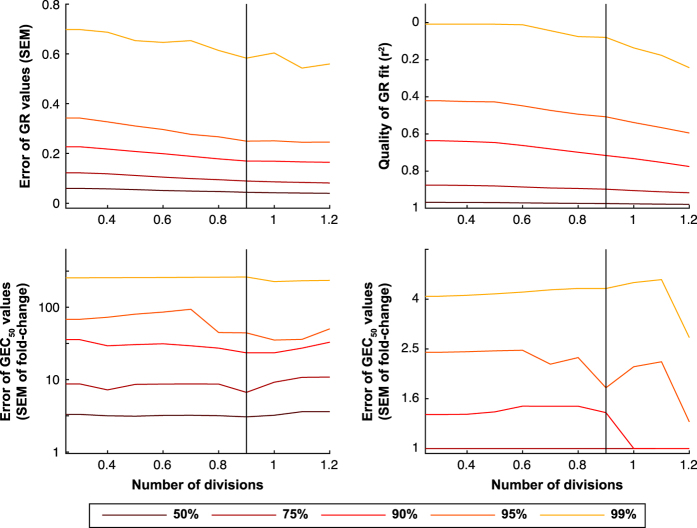
Data quality as a function of the cutoff in the number of cell divisions observed in parallel untreated cultures. Each plot shows the median, 75th-, 90th-, 95th- and 99th-percentiles for SEM of the GR values (top left), goodness of the sigmoidal fit (top right), *GEC*_*50*_ values (bottom left), and *GR*_*50*_ values (bottom right).

One of the key biological premises implicit in computing GR metrics is that cells in untreated wells are proliferating in a uniform, exponential, and continuous manner. In principle, this should be true for all established cell lines, but in a large-scale drug-response experiment it is inevitable that some variability will be encountered. For this reason, we discarded any plate in which control cells underwent fewer than 0.3 cell divisions. When we examined cell line/perturbagen pairs in which cells had undergone more than 0.9 divisions in the absence of drug, we observed a substantially lower standard error of the mean (SEM; Fig. 1). The 95th-percentile SEM was 0.35 for all cell line pairs tested and dropped to ~0.30 when we removed the 27% of the conditions that represent the slowest growing conditions (fewer than 0.9 divisions). We suspect that this arises because ratios between *x(c)*, *x*_*ctrl*_, and *x*_*0*_ are more sensitive to fixed measurement noise in the case of slow growing cultures. Low division number can arise because some cells are inherently slow growing, but sporadic examples of slow growth are more likely to reflect defects in the plate, excess evaporation of the medium, or pipetting error. Regardless, eliminating data for these slow growing conditions improved the SEM of the GR values and metrics.

The SEM of the GR values was below 0.1 for 75% of the data in dataset DS1, but 10% of the conditions in DS1 remained substantially variable across replicates (SEM > 0.2). Among the GR values with SEM > 0.2, we observed little bias by cell line. We therefore concluded that the data were impacted primarily by sporadic rather than systematic error (see Methods). With respect to estimation of drug potency, the SEM for *GR*_*50*_ across all perturbagens and cell lines was low (SEM below half an order of magnitude in drug concentration for 95% of the cell line/perturbagen pairs), but this estimate is biased by the many cases in which cells are resistant for which *GR*_*50*_ is set at infinity. For the subset of cell line/perturbagen pairs in which a substantial biological response is observed (*GR*_*50*_ <10 μM), 77% of the data have SEM values below half an order of magnitude. This is consistent with the *GEC*_*50*_ values, whose SEM is below an order of magnitude for 75% of the cell line/perturbagen pairs. Overall, the sigmoidal fit was good (r^2^>0.75) for 90% of the cell line/perturbagen pairs that show a substantial biological response (*GR*_*max*_<0.5). Taken together, these results show that, for this dataset, differences in GR value above 0.2 or in *GR*_*50*_ values above half an order of magnitude should reflect substantial differences in sensitivity.

## Usage Notes

**Figure 2:**
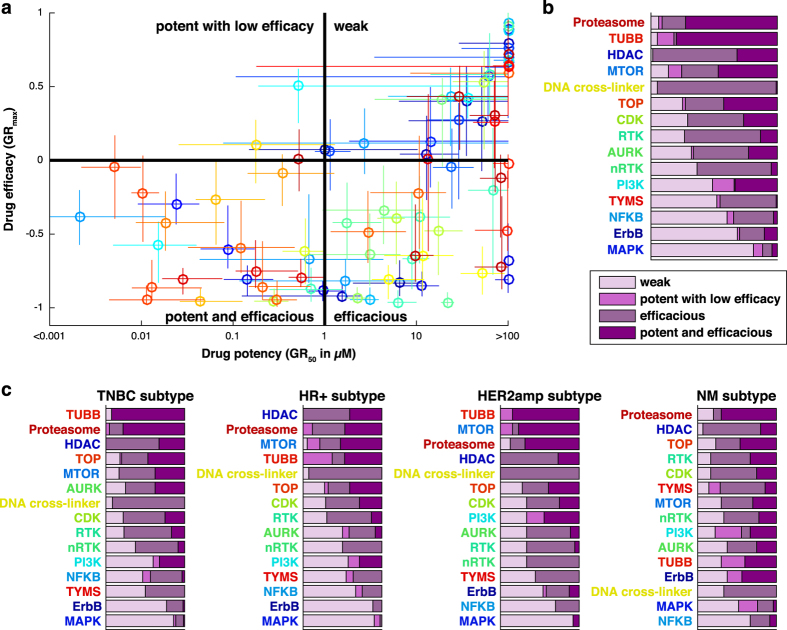
Relationship between efficacy (*GR*_*max*_ values) and potency (*GR*_*50*_ values). (**a**) Distribution of the *GR*_*50*_ values (x-axis) versus *GR*_*max*_ values (y-axis) by drug for all analyzed cell lines (averaged across biological replicates). (**b**,**c**) Distribution of the cell line/perturbagen pairs by drug class based on the response classes defined by efficacy (positive or negative *GR*_*max*_ value) and potency (*GR*_*50*_ value below or above 1 μM) for all cell lines (**b**) or separated by clinical subtype **(c**).

Uses of our data include identifying cell lines or subtypes that are particularly sensitive or resistant to a class of drugs (or the converse) for follow-up investigation. Using GR metrics for systematic pharmacogenomics studies has two advantages relative to standard approaches^2^: (1) it ensures that enrichment analysis is not biased by differences in division rate, and (2) it makes it possible to distinguish genes associated with drug-induced cytotoxicity (negative *GR*_*max*_ values) from cytostasis (*GR*_*max*_ values between 0 and 1). In the current study, we first compared potency, as measured by *GR*_*50*_ values, and efficacy, as measured by *GR*_*max*_ values, by drug class across cell lines (see Table 1 (available online only) for annotations; Fig. 2 and DS3 [Data Citation 2]). This showed that drugs targeting tubulin and the proteasome are among the most potent (low *GR*_*50*_), whereas HDAC inhibitors and DNA cross-linkers have the highest efficacy (low *GR*_*max*_). Many drugs induced a cytotoxic response at the highest concentrations (negative *GR*_*max*_ values), but most MAPK inhibitors and the majority of the ErbB, PI3K, and RTK inhibitors had positive *GR*_*max*_ values, showing that such drugs are partially cytostatic in most breast cancer cell lines. When results were sorted by clinical subtype, we found, for example, that tubulin-binding drugs were generally less efficacious in non-malignant (NM) and hormone-receptor-positive cell lines (HR^+^) than in HER2-amplified (HER2^amp^) and triple-negative (TNBC) cells; such drugs are used most widely in the treatment of TNBC.

**Figure 3:**
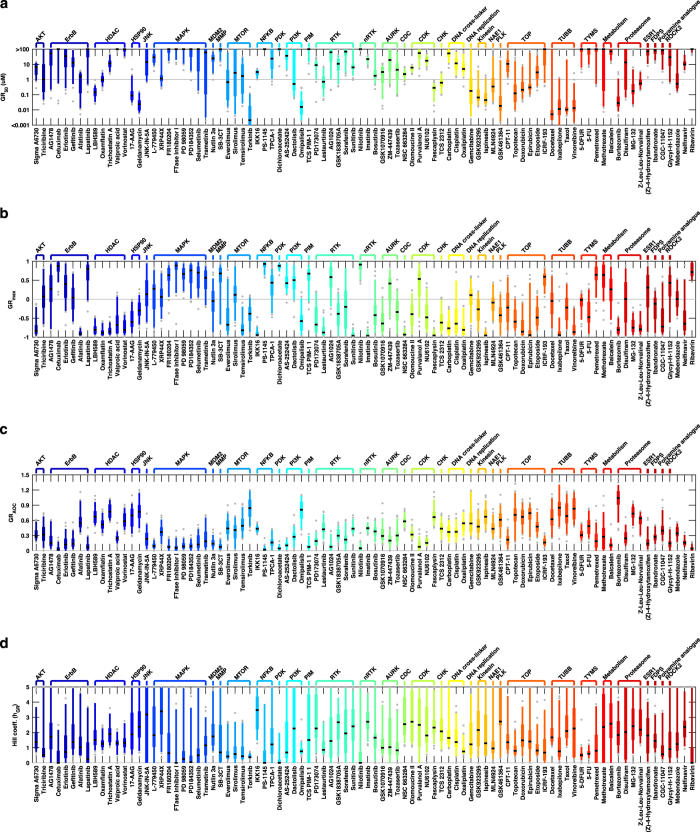
Distribution of the GR metrics by drug for all analyzed cell lines (averaged across biological replicates). (**a**) *GR*_*50*_ values. (**b**) *GR*_*max*_ values. (**c**) *GR*_*AOC*_. (**d**) Hill coefficient (*h*_*GR*_).

Contemporary analysis of tool compounds and therapeutic drugs focuses almost entirely on comparing differences in drug potency, with relatively little attention paid to efficacy as measured by maximum drug effect. We have previously shown, however, that both *IC*_*50*_ and *E*_*max*_ values for the Heiser et al.^8^ dataset are highly variable across drugs and cell lines^13^. When we repeated this analysis using the expanded dataset and GR-based drug metrics described in this work, we came to the same conclusion, namely that values for *GR*_*50*_, *GR*_*max*_, *GR*_*AOC*_, and *h*_*GR*_ are variable across cell lines and drugs (Fig. 3). For some drugs, predominantly MAPK and ErbB inhibitors, *GR*_*50*_ and *GR*_*max*_ values were correlated, but further analysis showed that this arose simply because a few cell lines were drug sensitive whereas the majority were resistant. When we examined the subset of cell line/perturbagen pairs that resulted in cytotoxicity (*GR*_*max*_<0), we observed no correlation between *GR*_*50*_ and *GR*_*max*_ (Spearman’s p > 0.05).

**Figure 4:**
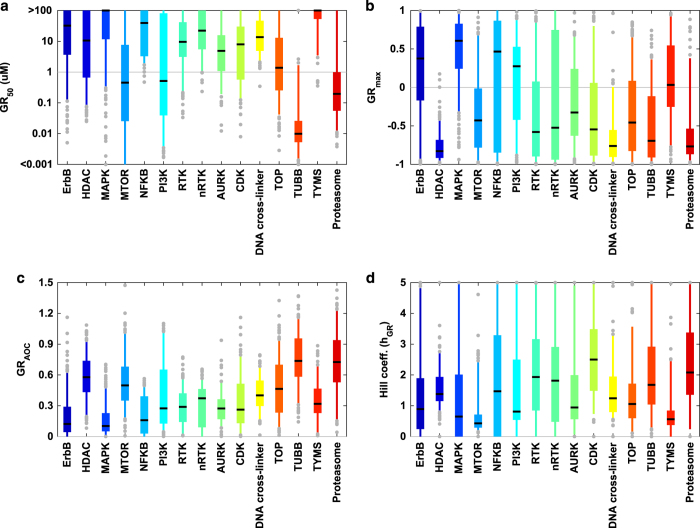
Distribution of the GR metrics by drug class for all analyzed cell line/perturbagen pairs (averaged across biological replicates). (**a**) *GR*_*50*_ values. (**b**) *GR*_*ma*x_ values. (**c**) *GR*_*AOC*_. (**d**) Hill coefficient (*h*_*GR*_).

Aggregating drugs by class (based on nominal targets–see Table 1 ((available online only) for these annotations; Fig. 4 and DS4 [Data Citation 2]) also revealed systematic variation in efficacy, potency, and Hill coefficient by drug class. For example, *GR*_*50*_ values for tubulin-binding drugs are very similar across cell lines; variation in response primarily involves *GR*_*max*_ values, many of which are negative (Figs 3a,b and 4a,b). mTOR and PI3K inhibitors as a class have very shallow dose-response curves characterized by low Hill coefficients (*h*_*GR*_; Figs 3d and 4d) and positive *GR*_*max*_ values, reflecting partial growth inhibition. Area over the dose-response curve (*GR*_*AOC*_; Figs 3c and 4c) can also be used to assess drug potency and efficacy in a single metric, but we have shown that *GR*_*AOC*_ values have lower information content than *GR*_*50*_ and *GR*_*max*_ values taken jointly^2^ because the latter two are biologically different. We therefore propose that future studies of cellular factors, genetic changes, and epigenomic variants controlling the responsiveness of cells to anti-cancer drugs consider associations with *GR*_*50*_, *GR*_*max*_, and *h*_*GR*_ independently.

## Additional information

**How to cite this article:** Hafner, M. *et al.* Quantification of sensitivity and resistance of breast cancer cell lines to anti-cancer drugs using GR metrics. *Sci. Data* 4:170166 doi: 10.1038/sdata.2017.166 (2017).

**Publisher’s note:** Springer Nature remains neutral with regard to jurisdictional claims in published maps and institutional affiliations.

## Supplementary Material



## Figures and Tables

**Table 1:** List of perturbagens in dataset DS1 and assigned drug class.

**Drug**	**Drug class**
(Z)-4-Hydroxytamoxifen	N/A
17-AAG	HSP90
2-deoxyglucose	N/A
5-DFUR	TYMS
5-FU	TYMS
AG1024	RTK
AG1478	ErbB
AS-252424	PI3K
Afatinib	ErbB
Baicalein	Metabolism
Bortezomib	Proteasome
Bosutinib	nRTK
Bromopyruvic acid	Metabolism
CGC-11047	N/A
CPT-11	TOP
Carboplatin	DNA cross-linker
Celecoxib	COX
Cetuximab	ErbB
Chk2 inhibitor II	CHK
Chloroquine	N/A
Cisplatin	DNA cross-linker
Dichloroacetate	PDK
Disulfiram	Proteasome
Docetaxel	TUBB
Doxorubicin	TOP
Epirubicin	TOP
Erlotinib	ErbB
Etoposide	TOP
Everolimus	MTOR
FR180204	MAPK
FTase Inhibitor I	MAPK
Fascaplysin	CDK
GM6001	MMP
GSK1070916	AURK
GSK461364	PLK
GSK923295	Kinesin
GW-5074	MAPK
GW843682X	PLK
Gefitinib	ErbB
Geldanamycin	HSP90
Gemcitabine	DNA replication
Glycyl-H-1152	N/A
ICRF-193	TOP
IKK16	NFKB
Ibandronate	N/A
Imatinib	nRTK
Ispinesib	Kinesin
Ixabepilone	TUBB
JNK-IN-5A	JNK
L-779450	MAPK
LBH589	HDAC
LY294002	PI3K
Lapatinib	ErbB
Lestaurtinib	RTK
MG-132	Proteasome
MLN4924	NAE1
Mebendazole	N/A
Methotrexate	Metabolism
Methylglyoxal	N/A
NSC 663284	CDC
NU6102	CDK
Nelfinavir	N/A
Nilotinib	nRTK
Nutlin 3a	MDM2
Olomoucine II	CDK
Oxaliplatin	DNA cross-linker
Oxamflatin	HDAC
PD 98059	MAPK
PD173074	RTK
PD184352	MAPK
PS-1145	NFKB
Pemetrexed	TYMS
Pertuzumab	ErbB
Pertuzumab	N/A
Purvalanol A	CDK
QNZ	NFKB
Ribavirin	N/A
Ro 32-0432	PKC
SB-3CT	MMP
Selumetinib	MAPK
Sigma A6730	AKT
Sorafenib	RTK
Sulindac sulfide	COX
Sunitinib	RTK
TAPI-0	N/A
TCS 2312	CHK
TCS PIM-1 1	PIM
TPCA-1	NFKB
Taxol	TUBB
Temsirolimus	MTOR
Topotecan	TOP
Tozasertib	AURK
Trametinib	MAPK
Trastuzumab	ErbB
Trichostatin A	HDAC
Triciribine	AKT
U-0126	MAPK
Valproic acid	HDAC
Vinorelbine	TUBB
Vorinostat	HDAC
XRP44X	MAPK
Z-Leu-Leu-Norvalinal	Proteasome
ZM-447439	AURK
Dactolisib	PI3K
GSK1838705A	RTK
Omipalisib	PI3K
Torkinib	MTOR
Sirolimus	MTOR
